# Recommendation for response to the COVID-19 pandemic: Korean context of “distancing in daily life,” considering vulnerable population

**DOI:** 10.1186/s12939-020-01309-x

**Published:** 2020-11-06

**Authors:** Ja Young Kim, Jin-Ok Han, Heeyoung Lee

**Affiliations:** 1 Gyeonggi Public Health Policy Institute, 7th Floor, 172, Dolma-ro, Seongnam-si, Gyeonggi-do 13605 Republic of Korea; 2 Seoul National University Bundang Hospital, 82, Gumi-ro 173 Beon-gil, Seongnam-si, Gyeonggi-do 13620 Republic of Korea

**Keywords:** Vulnerable population, Vulnerable group, COVID-19, Personal quarantine

## Abstract

While the coronavirus disease 2019 (COVID-19) pandemic is an ongoing worldwide, including South Korea (hereinafter Korea), it is impossible to predict the duration of the pandemic. To stop the spread of COVID-19, “social distancing,” which included mandatory lockdown, and attention to personal hygiene are being adopted globally as non-pharmaceutical preventive strategies. In Korea, after maintaining strong social distancing rules for a while, the government transitioned to implementing “distancing in daily life” since May 6, 2020. The distancing in daily life was combined with infection prevention activities to stop the COVID-19 pandemic, while guaranteeing one’s daily life and economic activities.

In this regard, the Ministry of Health and Welfare in Korea disclosed key rules for personal quarantine. The five key rules for individual infection control are as follows: to stay at home for 3–4 days if you feel unwell, keep a distance of two arms’ length from others, to wash your hands for 30 s and cough or sneeze into your sleeves, ventilate at least twice a day and disinfect regularly, and stay connected while physically distancing. However, for vulnerable populations, it is very difficult to follow such rules.

Thus, we attempted to recommend how the society could support such vulnerable populations who may face difficulties in following these individual infection control rules. Through our recommendations for the weakest part of our society, we expect to strengthen the overall social structure.

## 요약

전세계적으로 코로나19 관련 상황이 장기화되고 있고, 우리나라 역시 종식 시점에 대한 전망이 어렵다. 그럼에도 강력한 사회적 거리두기를 지속하기에는 어려움이 있기 때문에 국내에서는 5월 6일부터 생활 속 거리두기 단계로 전환하였다. 이러한 생활 속 거리두기는 일상생활과 경제활동을 보장하면서, 코로나19 유행 차단을 위한 감염예방 활동이 조화되는 방향으로 전개 중이었다. 보건복지부에서는 이와 관련하여 개인방역 핵심 수칙 등을 공개하였다.

발표된 개인방역 핵심 수칙은 ‘아프면 3 ~ 4일 집에 머물기’, ‘사람과 사람 사이, 두 팔 간격 거리두기’, ‘30초 손씻기, 기침은 옷소매에’, ‘매일 2번 이상 환기, 주기적 소독’, ‘거리는 멀어져도 마음은 가까이’ 이렇게 5가지로 구성되었다. 하지만, 우리 주변의 취약계층에 속한 대상자들은 이러한 생활수칙을 준수하는데 어려움이 따를 수밖에 없다.

이에 생활 속 거리두기 실천을 위한 개인방역 핵심 수칙을 바탕으로 우리 사회의 취약계층별로 직면하게 되는 어려움과 이들을 위한 지원방안을 제시하고자 하였다. 이러한 노력을 통해 우리 사회의 가장 약한 부분을 돌아보게 되어 사회 구조 전반을 강화할 수 있을 것으로 기대되는 바이다.

## Background

On March 11, 2020, the World Health Organization (WHO) officially declared that the outbreak caused by coronavirus disease 2019 (COVID-19) as a pandemic [1], and since then, the situation has been prolonged, with the overall number of confirmed cases increasing continuously. In Korea, the total cumulative number of confirmed cases is 24,988 as of October 15, 2020, and of which 23,082 cases have been discharged from isolation, and 52 cases has increased compared to the previous day [2]. To date, various efforts have been made to develop vaccines/therapeutic drugs, and it is still difficult to predict the end of COVID-19.

As a response to the COVID-19 pandemic, “social distancing” is one of the non-pharmaceutical preventive strategies for slowing down the spread of the virus effectively; thus, it is a vital long-term approach. In addition, various ways of “social distancing” have been implemented worldwide, including mandatory lockdowns and paying attention to personal hygiene [3, 4]. Even though Korea did not employ the lockdown strategy, the government had required people to keep strong social distances for about a month (from March 22 to April 19). After that, the government has implemented “Distancing in daily life” since May 06, 2020 [5].

In Korea, a “distancing in daily life” strategy has been used for curbing the spread of COVID-19 in the long-term while maintaining social interaction. The strategy is based on the values that all members of the society are responsible for overcoming the COVID-19 pandemic and protecting their own health. The main goals of ‘distancing in daily life’ are as follows: prevent virus infiltration into living spaces, minimize conditions that are favorable for pathogen transmission and survival, minimize discharge of the virus outside the body, and trace and block the virus transmission path.

The infection control strategy consists of five key rules and four supplementary actions for individuals [5].

- Five key rules for individual infection control
  - Rule 1: “Stay home for 3 to 4 days if you feel unwell.”
  - Rule 2: “Keep a distance of two arms’ length from others.”
  - Rule 3: “Wash your hands for 30 seconds. Cough or sneeze into your sleeve.”
  - Rule 4: “Ventilate at least twice a day and disinfect regularly.”
  - Rule 5: “Stay connected while physically distancing.”
- Four Supplementary Actions for Individuals
  - Use of Masks
  - Disinfection of Environments
  - Guidelines for the Elderly and High-risk Groups
  - Healthy Lifestyle

Even though the strategy of “social distancing” was possible of making people feel exhausted and could lead to unavoidable economic recession in Korea, it was considered a successful model of “citizen participation in quarantine” due to voluntary actions of the public. Nevertheless, it was difficult for people to follow the rules. According to an online survey on the prevention of COVID-19 in daily life conducted by the Ministry of Health and Welfare in Korea, many concerns have been raised regarding the situation of vulnerable groups in the COVID-19 pandemic in keeping social distancing in daily life [2]. The Korean government is making efforts and taking actions, such as providing emergency relief funds and urgent support programs for unpaid leaves [6, 7]. Nevertheless, the vulnerable population is in need for further support.

The purpose of this article was to introduce the “distancing in daily life” strategy in Korea, provide tangible evidence for other countries facing the pandemic, and propose important considerations for the vulnerable population.

## Main text

As COVID-19 is a novel virus, we cannot understand fully the characteristics of the virus causing it and its impact on our health, but it is assumed that a specific population is more vulnerable to this pandemic than others [8]. It is difficult to define the term “vulnerable population” [9], but it generally includes foreign workers, homeless/poor urban residents, people with disabilities, older people, and so on.

### Foreign workers

Although the number of foreign workers, including illegal workers, is not known, the number of regular foreign workers under the Employment Permit System was reported to be 51,365 in 2019 [10], and over 70% of foreign workers in the Employment Permit System have been working in the manufacturing industry, which is known for its risky physical work environments. Usually, foreign workers perceive themselves to be legally unstable, and since they tend to share a single living space/residence with many people, they tend to live in poor conditions. Furthermore, their working environment is likely to be poor because over 70% of the foreign workers who work under the Employment Permit System in Korea [10] are engaged in the manufacturing industry, which is known to have a poor working environment. They also have limitations in accessing useful information due to language differences and can be excluded from the government’s public resource procurement system and benefit distribution.

### Homeless/poor urban residents

These people tend to have no place to live or live in an environment with a high risk of infection [11]. For example, homeless people often stay in shelters that are crowded and poor urban residents live in highly populated areas; thus, these groups are highly susceptible to the viral infection. Another serious problem is that the public hospitals they used to visit have been converted into public relief hospitals designated for handling COVID-19 cases; thus, their medical accessibility has reduced since the COVID-19 outbreak.

### People with disabilities

People with disabilities have limited physical or mental ability, depending on the disease severity. In-person services including community-based rehabilitation provided to them may have been stopped or limited during the COVID-19 pandemic. As they require continuous medical services, these combined problems could worsen their health status [12]. For instance, people with mental disabilities would feel more isolated and depressed during the COVID-19 pandemic than they did prior to it because the support services have been discontinued and contact with people has reduced. In addition, they have underlying medical conditions, such as high blood pressure and diabetes, and the pandemic may have worsened these problems.

### Older people

Older people aged over 60 years old have more underlying diseases than other age groups and have limited physical abilities [12]. In addition, the services provided to them by the public health center have been suspended or limited since the outbreak of COVID-19.

During the COVID-19 pandemic, it is crucial to take additional measures for vulnerable populations in terms of support provision and policy design. However, so far, the basic guidelines for distancing in daily life by the KDCA are mostly geared toward the general population and some high-risk populations with underlying diseases such as high blood pressure and diabetes. Therefore, in this commentary, we are going to examine the populations that would be at high risk of COVID-19 infection and what kind of support would be necessary for protecting them.

### Rule 1: “Stay home for 3-4 days if you feel unwell”

The KDCA recommends those feeling unwell to stay home for 3–4 days because COVID-19 patients with mild symptoms can spread the virus at an early stage [5]. To follow the first essential rule for individual infection control, it is necessary to let people use their sick leave when they have a fever or respiratory symptoms related to COVID-19. In the cases of confirmed diagnosis or close contact with COVID-19 patients, the Korean government tries to support sick pay for the isolation period. However, most people with fever or respiratory symptoms cannot receive this kind of support from the government.

Considering the unstable legal status of foreign workers, it is difficult for them to take days off or a sick leave in case of fever or respiratory symptoms before they receive a definitive diagnosis of COVID-19. Therefore, to help them adhere to the first key rule, the government should recommend companies to check the body temperature of the workers before they enter the workspace and provide support to the companies that provide preventive measures such as days off or sick leaves for those with fever or respiratory symptoms.

In addition, homeless people/poor urban residents often have unstable working conditions and thus resting itself can pose a threat to their survival. Thus, support may be needed in terms of special relief funds to maintain income during unemployment. In addition, it may be necessary to provide a thermometer to help them check their temperatures regularly and to provide a service they can visit only if they have difficulties in checking their own body temperature (people with disability, elderly people, etc.).

### Rule 2: “Keep a distance of two arms’ length from others”

According to government guidelines [5], COVID-19 is mainly transmitted through saliva droplets; thus, it is important to maintain 1 meter (or 2 arms’ length) gap between people. It is better to eat at home than going outside. However, in some cases, such as in the work environment, it would be very difficult to keep distance despite personal efforts. For example, in Korea, there was a COVID-19 outbreak among call center workers, and the number of cases rapidly increased due to the close distance working situation (call center cluster; [13]). Since then, Korea has recommended telecommuting (working from home), flexible work hours, etc. It may be necessary to control the population density or the number of people in a shared space to maintain proper distance. This change is only possible through cooperation between public and business sectors, rather than through individual efforts.

Foreign workers are more likely to work or live in population-dense environments, such as dormitories. Homeless or poor urban residents are likely to reside in crowded shelters or poor urban areas, and these environments make them vulnerable to the virus. Therefore, efforts to improve the residential environment are urgently needed, such as providing safe shelter.

### Rule 3: “Wash your hands for 30 seconds. Cough or sneeze into your sleeve”

This rule is relatively easier than other rules for everyone to practice and is the most basic method for minimizing COVID-19 transmission through contaminated hands or saliva droplets. However, it may not be easy for vulnerable populations to do this because their activities may be limited, but wearing masks can be helpful in following the rule. Thus, providing protective items, including masks, can be helpful.

In Korea, the government attempted to control the supply of masks, and for Korean residents, it was not challenging to buy masks. However, it was only recently that foreigners, including foreign workers, were able to get access to the public supply of masks. Those who do not have a foreigner registration card will not be able to buy masks through public supply systems. Thus, it would be imperative to implement a new policy to allow all foreign workers regardless of their legal status to have access to masks and quarantine items. In addition, basic preventive guidelines need to be offered in various languages. To help the homeless people or poor urban residents who do not have stable places to stay, hand sanitizers can be distributed in public places such as public restrooms and community centers. Since the risk of COVID-19 infection may be relatively high in these environments, masks and quarantine items need to be provided through various methods. For the disabled and older populations, the provision of masks and protective supplies is necessary because physical constraints can make it difficult for them to follow the rules and lead to poor access to relevant information. All provided information should be translated into sign language and braille to help those with low access to information about individual infection control. In fact, some local governments (Seoul Metropolitan Government) have set up a task force for supplying quarantine goods and implemented a project to visit vulnerable people and distribute masks and quarantine items such as hand sanitizers. This action should be followed by other local governments.

### Rule 4: “Ventilate at least twice a day and disinfect regularly”

The KDCA guidelines recommend ventilating at least twice a day to reduce the concentration of droplets containing COVID-19 virus and to disinfect surfaces on which infectious droplets could have landed [5]. It is important to manage the residential and workplace environments to the optimal, not only at the personal but also at the community level. In order to adhere to these rules, the ventilation system should be well maintained for regular ventilation (more than twice a day), and it is important to have relevant supplies for regular disinfection, which may be difficult to manage in some work and residential environments.

Foreign workers in crowded environments may find it difficult to change their situation by themselves. Therefore, disinfection of public spaces, such as foreign workers’ dormitories, may be crucial. For homeless people who do not have housing or residents of urban slums living in crowded environments, special services may be required, such as providing temporary shelter capable of quarantine management or visiting the areas to carry out disinfection/cleaning process. These visiting services may be equally necessary for the disabled and older people who are restricted in their daily life activities.

### Rule 5: “Stay connected while physically distancing”

Most vulnerable people could have been feeling isolated from society, and the COVID-19 pandemic must have made things worse for them. When “social distancing” is encouraged to curb the spread of the infection, it may be difficult to deal with mental problems alone given that direct support/service/visit is restricted. In particular, it may be difficult for them to cope with anxiety when exposed to sensational media reports about the COVID-19 pandemic situation coupled with no social interaction.

In Korea, so-called “psychological quarantine” is being primarily offered via psychological counseling for those in self-quarantine. However, psychological counseling services should also be provided to vulnerable populations who are socially isolated due to the COVID-19 pandemic. In fact, some local bodies are providing consultation services to older people by making regular phone calls and checking on their mental health.

## Conclusions

In this commentary, we examined the difficult conditions of vulnerable populations in implementing key rules of “distancing in daily life.” Vulnerable people are more likely to suffer from the COVID-19 pandemic than others. According to the WHO Regional Office for Europe report [14], strengthening social structures is essential for reducing socioeconomic damage. A strong social structure can help enhance social cohesion and participation and help prevent vulnerable people from falling behind. Therefore, effective protection/support policies are needed for vulnerable populations.

In particular, efforts should be made to provide an appropriate environment and educate on adequate preventive measures by organizing campaigns for individuals to follow the rules to prevent COVID-19 regardless of their social status. In fact, a budget of 15 billion Korea won was allocated to support unpaid workers, special employment, and freelancers. In addition to financial support, social support policies should be enacted for isolated vulnerable populations.

## Data Availability

Not applicable.

## Abbreviations

COVID-19: Coronavirus disease 2019
KDCA: Korea Disease Control and Prevention Agency
WHO: World Health Organization
